# Genomic sequence of the aflatoxigenic filamentous fungus *Aspergillus nomius*

**DOI:** 10.1186/s12864-015-1719-6

**Published:** 2015-07-28

**Authors:** Geromy G. Moore, Brian M. Mack, Shannon B. Beltz

**Affiliations:** United States Department of Agriculture, Agricultural Research Service, Southern Regional Research Center, New Orleans, LA USA

**Keywords:** *Aspergillus nomius*, Genome sequence, Gene ontology, Phylogenomics, Mating-type locus

## Abstract

**Background:**

*Aspergillus nomius* is an opportunistic pathogen and one of the three most important producers of aflatoxins in section *Flavi*. This fungus has been reported to contaminate agricultural commodities, but it has also been sampled in non-agricultural areas so the host range is not well known. Having a similar mycotoxin profile as *A. parasiticus*, isolates of *A. nomius* are capable of secreting B- and G- aflatoxins.

**Results:**

In this study we discovered that the *A. nomius* type strain (NRRL 13137) has a genome size of approximately 36 Mb which is comparable to other Aspergilli whose genomes have been sequenced. Its genome encompasses 11,918 predicted genes, 72 % of which were assigned GO terms using BLAST2GO. More than 1,200 of those predicted genes were identified as unique to *A. nomius*, and the most significantly enriched GO category among the unique genes was oxidoreducatase activity. Phylogenomic inference shows NRRL 13137 as ancestral to the other aflatoxigenic species examined from section *Flavi*. This strain contains a single mating-type idiomorph designated as *MAT1-1*.

**Conclusions:**

This study provides a preliminary analysis of the *A. nomius* genome. Given the recently discovered potential for *A. nomius* to undergo sexual recombination, and based on our findings, this genome sequence provides an additional evolutionary reference point for studying the genetics and biology of aflatoxin production.

## Background

*Aspergillus nomius* is an aflatoxigenic fungal species, grouped in the genus’ section *Flavi*, which is often overshadowed by the more readily-sampled aflatoxigenic species such as *A. flavus* and *A. parasiticus*. The relatively little research attention applied to *A. nomius* likely relates to a historical belief that its occurrences are rare [1, 2]; however, more recent reports suggest that this fungus has a wider ecological distribution and a greater economic potential than once believed [3, 4]. Most samplings of *A. nomius* have been reported from infected insects [5–8] and non-agricultural soils [4]. Reports also exist that show *A. nomius*’ potential as a human pathogen [9] as well as its indirect potential for animal and human impact through aflatoxin contamination of agricultural commodities such as maize [4], wheat [6], sugar cane [10], and tree nuts [6, 11].

In culture, *A. nomius* appears similar to the *A. flavus* L morphotype (L-type), so differentiation between these closely-related species can be fairly subjective and open to misidentification. The *A. flavus* L-type produces fewer large sclerotia and abundant conidia while the *A. flavus* S-type produces abundant small sclerotia and fewer conidia, but these are not considered different species [12]. Historically, aflatoxin profiles have been used to distinguish between *A. flavus* and *A. nomius* since their macro- and micro-morphologies were highly similar. If an isolate produced only B-aflatoxins it was classified as *A. flavus*, and if B- and G- aflatoxins were produced, it was considered to be *A. nomius*. Whereas *A. parasiticus* has several distinct morphological characteristics, this is not the case for distinguishing between *A. flavus* and *A. nomius*. For example, *A. parasiticus* typically produces asexual spore forming structures (conidiophores) with a single layer of spore-forming cells (phialides) and lacking an intermediate layer of cells (metulae), and has dark green conidia which are noticeably rough-walled [13]. These differences are not observed when comparing *A. flavus* and *A. nomius* whose colony diameters and coloration are very similar, tending towards yellow-green on Czapek’s Dox (CZ) agar. The size and texture (smooth to finely roughened) of their conidia are also very similar. The sclerotia of *A. nomius* are reported to be more elongated than those of *A. flavus*, and it has been observed that *A. flavus* grows well at 42 °C while *A. nomius* growth is slowed [6]. Due to the number of similarities between *A. flavus* and *A. nomius*, genomic comparisons may be more useful than traditional methods for species identifications.

There have been additional cryptic aflatoxin producing species recently characterized that are related to, and potentially indistinguishable from, *A. flavus* (L- or S-type), *A. nomius* and/or *A. parasiticus* such as *A. arachidicola*, *A. minisclerotigenes* and *A. parvisclerotigenus* [14–16]; therefore, a holistic approach to fungal identification and classification is required to make certain what is being characterized is not a naturally-occurring mutant or a hybrid offspring exhibiting a recombinant phenotype. This will involve comparisons of species morphology, extrolite production, host/niche range, and genomics [17]. A population study of *A. nomius* isolates sampled throughout Thailand revealed three potential clades or lineages for this species [3]. Within the last few years the sexual (teleomorphic) state was discovered and assigned to the genus *Petromyces*, and although the fungus is heterothallic, some strains contain both mating-type (MAT) idiomorphs and exist as functionally bisexual- meaning that either idiomorph is capable of outcrossing [4]. Its bisexuality may allow for more rampant sexual recombination in the environment regardless of the dominant mating type present. With the potential for hybridization between aflatoxigenic species (unpublished observations), *A. nomius* might serve as a mycotoxin reservoir in non-agricultural areas that are near agricultural fields [3], and assist in the generation of offspring (i.e., new ‘species’) with greater host ranges and novel chemotype profiles.

In this study we sequenced the genome of *A. nomius* to improve our understanding of its evolutionary place among the aflatoxigenic fungi, to ascertain those features that distinguish it from other closely-related species, and to promote research interest for this organism since it may offer more insights into the speciation and development of novel aflatoxigenic species.

## Results and discussion

### Genome details for *A. nomius* NRRL 13137

We sequenced the genome of *A. nomius* type strain NRRL 13137 to 42x coverage. This gave us 5 million reads with a median read length of 306 bp, 2,713 contigs of data (950 of which were ≥ 1000 bp), and a N50 length of 66,657 bp. The raw sequence reads have been deposited in the NCBI Sequence Read Archive as SRR1297207. The genome assembly of *A. nomius* NRRL 13137 is approximately 36 Mb in size and includes 11,918 protein-encoding genes (Table 1). Comparatively, *A. nomius* has a similar genome size to the other sequenced species from section *Flavi*. For example, assemblies for *A. flavus* NRRL 3357 and *A. oryzae* RIB40 are approximately 37 Mb [18, 19]. Table 2 shows various morphological, toxigenic, and genomic differences between *A. flavus*, *A. nomius* and *A. parasiticus*. Reports of the genome sizes of other sequenced *Aspergillus* species indicate that *A. niger* CBS 513.88, *A. nidulans* FGSC A4, and *A. fumigatus* Af293 have genome assemblies that include approximately 34 Mb, 30 Mb, and 29 Mb, respectively [20–22]. The reason for section *Flavi* species having larger genomes than other sequenced Aspergilli remains uncertain, but one reported possibility is gene acquisition [18]. The *A. oryzae* genome is one of the larger genomes of the section *Flavi* examined, but it also has a higher percentage of repetitive DNA than the others (Table 2). Of the nearly 12,000 predicted genes for *A. nomius*, 39 % (4,674) were assigned as hypothetical with no functional annotation. Although similar numbers of predicted genes were found in other sequenced species, due to limits in sequencing the *A. nomius* genome it should be noted that genes may not have been annotated if they happened to exist between contigs that have no overlap. The number of genes considered unique to *A. nomius* is 1,124 or only about 9 % of the total number of its predicted genes. SMURF* predicted *A. nomius* having an estimated 44 secondary metabolite clusters (SMCs) while antiSMASH predicted 53 SMCs (Table 3). The difference between these counts is likely related to stringency of predictions between the two programs; however, it is still recommended that both tools be used since they incorporate different algorithms in cluster prediction [23]. For example, SMURF* predictions include DMAT backbone genes whereas antiSMASH does not, but even without considering DMAT clusters antiSMASH predicted a greater number of SMCs than SMURF*. Beyond section *Flavi*, our 44 SMURF*-predicted SMCs in *A. nomius* was comparable to the 40 in *A. nidulans*, and noticeably higher than the 25 in *P. chrysogenum*. Andersen et al. reported a putative count of 51 SMCs in *A. nidulans* which was also based on SMURF* but may have also included “-like” genes (e.g., NRPS-like) [24]. According to antiSMASH predictions, *A. nomius* had a much greater number of SMCs than both *A. nidulans* (35) and *P. chrysogenum* (31). *Aspergillus parasiticus* had the most predicted SMCs across all Aspergilli examined, based on predictions from both programs, while *A. nidulans* had the least number of SMCs (Table 3).

**Table 1:** Genome characteristics for the *A. nomius* type strain NRRL 13137

Genome characteristic	Value
General	
Assembly size (bp)	36,156,695
G + C (%)	49
Protein coding genes	11,918
Protein coding genes >100 amino acids	11,672
Predicted protein coding sequences >100 amino acids	
Coding (%)	49
Gene density (1 gene every n bp)	3,034
Median gene length (bp)	1,447
Mean gene length (bp)	1,732
Average number of exons per gene	3.4

**Table 2:** Morphological, phenotypic and genomic comparison of *A. flavus*, *A. nomius* and *A. parasiticus*

Species	Morphology		Phenotype	Genomics			
	Macro^a^	Micro^b^	Toxic SMs^c^	Size (Mb)^d^	Genes^e^	GC (%)^f^	Rep. DNA (%)^g^
*A. flavus* L	55–65 mm velvety to floccose olive green sclerotia (l + v)	Radiate to columnar 400–800 μm, rf/fr 20–45 μm, gl/el u/b 3–6 μm, gl/el, sm/fr	B_1_, B_2_, CPA	36.89	13,485	48.22	1.17
*A. nomius*	40–70 mm velvety to floccose olive green sclerotia (e)	Radiate 300–1100 μm, rf/fr 25–65 μm, gl/el u/b 4.5–6.5 μm, gl/el, sm/fr	B_1_, B_2_, G_1_, G_2_	36.14	11,914	48.86	1.09
*A. parasiticus*	45–65 mm velvety to floccose dark green sclerotia (o)	Radiate 250–500 μm, fr/rf 20–35 μm, gl/el u/b 3.5–6 μm, gl, rf	B_1_, B_2_, G_1_, G_2_, OMST	39.82	13,543	47.72	1.40

^a^Colony characters on Czpaek’s medium, incubated at 25 oC for 7 days: diameter; texture; color. Sclerotia large and variable in shape (l + v), elongate (e), or occasionally formed (o)

^b^Conidiophore characters: conidial head; stipe (rough = rf, finely-roughened = fr); vesicle (globose = gl, elongate = el); seriation (uniseriate = u, biseriate = b, both/either = u/b); conidia (globose = gl, elongate = el, smooth = sm, finely-roughened = fr, rough = rf)

^c^Major toxic secondary metabolites: B and G aflatoxins; cyclopiazonic acid (CPA); O-methylsterigmatocystin (OMST)

^d^Approximate sizes of sequenced genomes

^e^Estimated gene counts based on annotation

^f^GC content for each genome

^g^Percentage of repetitive DNA

**Table 3:** Putative secondary metabolite clusters within various species included in our phylogenomic examination (Fig. 2)

Species	SMURF^*^ analysis^a^					antiSMASH^b^			
	NRPS	PKS	NRPS-PKS	DMAT	Total	NRPS	PKS	NRPS-PKS	Total
*A. flavus* L	14	24	2	7	47	18	24	3	45
*A. flavus* S	11	22	3	8	44	17	30	4	51
*A. nidulans*	10	25	1	4	40	11	22	2	35
*A. nomius*	14	20	3	7	44	17	28	8	53
*A. oryzae*	12	22	1	7	42	18	28	2	48
*A. parasiticus*	17	22	1	8	48	21	32	2	55
*P. chrysogenum*	8	15	1	1	25	10	19	2	31

^a^SMURF^*^ predictions do not include “-like” backbone genes (NRPS-like, PKS-like, NRPS-PKS-like, DMAT-like)

^b^antiSMASH software does not consider DMAT backbone genes and its predictions do not include “-like” backbone genes

### Gene ontology and enrichment for *A. nomius* NRRL 13137

A total of 8,599 genes were assigned gene ontology (GO) terms. Level-3 GO distribution results for each domain revealed that most gene products within the cellular component domain are involved in cell, membrane and organelle structure; within the biological process domain, most genes are involved in metabolic, cellular and single-organism processes; and analysis of the molecular function domain revealed that the greatest numbers of *A. nomius* genes are involved in ion binding, organic cyclic compound binding and heterocyclic compound binding (data not shown). For the 1,264 unique *A. nomius* genes, 13 of the 19 most significantly-enriched GO terms as shown by the Fisher’s Exact test were associated with molecular function, and six were associated with biological process. Significance levels, however, indicated that sequences associated with oxidoreductase activity had the highest enrichment (*p*-value = 9.67E-11) of all the GO terms/categories (Table 4). There were 227 protein sequences that were assigned to the “oxidoreductase activity” category of the molecular function domain. Of these proteins, only 43 were assigned functional annotation with 102 identified as hypothetical. The greatest number of any particular protein within the category of oxidoreductase activity was related to cytochrome P450 (n = 14). The epoxide metabolic process, and both fumagillin processes listed in Table 4, shared the same quantities and types of proteins (n = 12) which would indicate overlapping functions for these biological processes. Of the 255 proteins affiliated with the molecular function of heme binding, 66 were identified as hypothetical and only 21 of these were assigned functional annotation. As with oxidoreductase activity, the greatest number of any particular protein within the category of molecular function was related to cytochrome P450 (n = 93). Cytochrome P450 enzymes in fungi contribute to a multitude of complex bioconversions which are necessary for their adaptation and survival on various substrates [25].

**Table 4:** Fisher’s exact test examining GO term enrichment for genes unique to *A. nomius*

GO term	*P*-Value
Oxidoreductase activity	9.67E–11
Epoxide metabolic process	3.55E–09
Fumagillin biosynthetic process	3.55E–09
Fumagillin metabolic process	3.55E–09
Heme binding	3.92E–08
Tetrapyrrole binding	4.50E–08
Ether biosynthetic process	1.23E–07
Iron ion binding	5.35E–07
Catalytic activity	1.17E–06
Transition metal ion binding	1.95E–06
Oxidation-reduction process	5.03E–06
Modified amino acid binding	8.85E–06
Oxidoreductase activity^a^	1.11E–05
Ether metabolic process	1.21E–05
Phosphopantetheine binding	1.94E–05
Ion binding	2.68E–05
Amide binding	4.10E–05
Vitamin binding	9.00E–05
Amino acid binding	9.63E–05

^a^Acting on paired donors, with incorporation or reduction of molecular oxygen

### Comparison of annotated genes and phylogenomics among sequenced *Aspergillus* genomes

**Fig. 1:**
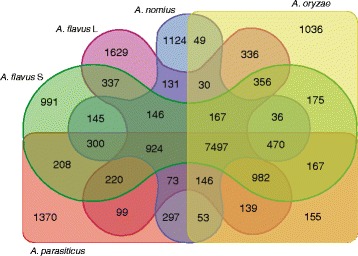
A Venn diagram quantifies unique and orthologous genes for multiple species in section *Flavi.* This diagram of overlapping shapes includes species names, gene counts, and color-shading: NRRL 13137 (*A. nomius*) is shaded blue, NRRL 3357 (*A. flavus* L) is shaded purple, AF70 (*A. flavus* S) is shaded green, SU-1 (*A. parasiticus*) is shaded red, and RIB40 (*A. oryzae*) is shaded yellow

An orthology analysis (Fig. 1) revealed that the five species examined from section *Flavi* share 7,497 genes. When *A. nidulans* and the outgroup taxa, *P. chrysogenum*, were added to the analysis the number of shared genes decreases to 4,977 (which were all used as input for our phylogenomic analysis). The count of 1,124 unique genes in *A. nomius* is not the highest number for the five section *Flavi* species examined, however, since *A. flavus* L-type (NRRL 3357) and *A. parasiticus* (SU-1) appear to harbor more unique genes (1,629 and 1,370, respectively). *Aspergillus oryzae* (RIB40) has 1,036 unique genes while S-type *A. flavus* (AF70) has the fewest unique genes (n = 991). The highest quantity of shared unique genes between *A. nomius* and each of the other species examined is with *A. parasiticus* (n = 297), and the lowest quantity shared is with *A. oryzae* (n = 49). The number of shared unique genes among *A. flavus* L, *A. parasiticus* and *A. nomius* is less than 1 % (n = 73) compared to those shared by all section *Flavi* species examined; however, the inclusion of another aflatoxin producer (*A. flavus* S) increased the number of shared unique genes by 11 % (n = 924). The finding that *A. flavus* S contained many of the unique genes found in the other aflatoxigenic species examined warrants further investigation since it shares more unique genes with three species that, among themselves, do not share as many (Fig. 1). The fewest numbers of shared genes involved an overlap of *A. nomius* and *A. oryzae* with *A. parasiticus* (n = 53), *A. flavus* L (n = 36), and *A. flavus* S (n = 30). Although closer examination of the unique genes within these closely-related species will result in better delimitation, it should be considered that the unique genes that separate *A. flavus* L, *A. flavus* S, and *A. oryzae* from one another may be more strain-specific than species-specific. Additionally, a closer examination of the unique genes within these fungi could offer a more refined diagnostic PCR methodology to aid in species delimitation.

**Fig. 2:**
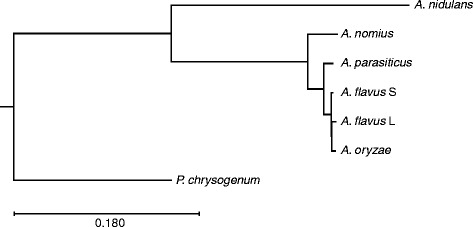
Phylogenomic comparison of sequenced *Aspergillus* species reveals patterns of ancestry. This tree was inferred from phylogenomic comparisons of multiple *Aspergillus* species (*A. nidulans*, *A. nomius*, *A. parasiticus*, *A. flavus* L, *A. flavus* S and *A. oryzae*) with *Penicillium chrysogenum* as the outgroup taxa

Phylogenomic associations were used to infer a tree for multiple Aspergilli (Fig. 2). Using *Penicillium chrysogenum* as the outgroup taxa, we observed a divergence of the *A. nidulans* genome from those of section *Flavi*, which was expected. Although exhibiting a great deal of overall genomic similarity, *A. nomius* was observed to be the most basal genome of the species examined from section *Flavi*, followed by the *A. parasiticus* (SU-1) genome and then that of *A. flavus* S (AF70). The *A. flavus* L (NRRL 3357) and *A. oryzae* (RIB40) genomes appear to have diverged most recently. Based on our phylogenomic inference, *A. nomius* is confirmed as the ancestral organism compared to the other section *Flavi* species examined, and would suggest that it may be the progenitor to the other aflatoxigenic species in section *Flavi*. Previous conventional phylogenetic studies have given researchers an idea of the evolutionary relationships for the Aspergilli; therefore, a phylogenomic comparison including the genomes of all aflatoxigenic species would offer better resolution for the evolution of the aflatoxigenic phenotype. Similarly, phylogenomic comparison of the remaining species from section *Flavi* (e.g., *A. alliaceus*) would offer more insights into the relatedness of these fungi. For example, *A. alliaceus* is usually observed basal to the other species in many phylogenetic inferences involving section *Flavi* [15], but these are usually based on analyses of one to three individual loci. Ehrlich et al. [3] reported the possibility of multiple lineages of *A. nomius* that include genetically-distinct isolates, so a comparative phylogenomic study of strains from each lineage may ascertain whether or not these strains should all be considered *A. nomius*.

### *A. nomius* NRRL 13137 MAT locus investigation revealed a single *MAT1-1* idiomorph

**Fig. 3:**
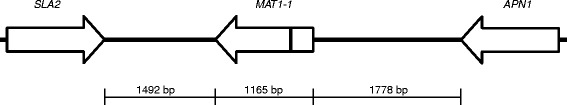
The mating-type locus of *A. nomius* NRRL 13137 contains a single *MAT1-1* idiomorph. This schematic diagram for the mating-type locus of NRRL 13137 shows the orientation and physical distance shared among the *MAT1-1* idiomorph and the adjacent upstream and downstream genes. The black vertical line in the *MAT1-1* arrow represents the approximate location of a 52 bp intron

According to Horn et al. [4], *A. nomius* is a heterothallic fungus that is incapable of self-fertilization despite some observed strains possessing both idiomorphs. The *A. nomius* type strain, NRRL 13137, is considered a *MAT1-1* strain because only a single α-box idiomorph has been shown to be present using diagnostic PCR [26], and our sequencing results support this finding. BLAST query of nucleotide and protein sequences, for a *MAT1-2* idiomorph, to the NRRL 13137 genome failed to uncover any homologs. The *MAT1-1* gene of NRRL 13137 spans 1,165 bp, and is comprised of two exons adjoined by a single 52 bp intron. This MAT gene is flanked by two other genes, *SLA2* and *APN1*, which have respective lengths of 4,138 bp and 2,189 bp, and are separated from the MAT gene by nucleotide distances of 1,492 bp and 1,778 bp, respectively (Fig. 3). These two flanking genes appear to have conserved locations upstream and downstream of the MAT genes in other species from section *Flavi* [26]. According to Metzenberg and Glass, MAT idiomorphs within heterothallic fungi will occupy a similar chromosomal location [27]; for *A. oryzae*, *A. flavus* and *A. parasiticus*, the idiomorphs are reported to reside on chromosome VI [26]. We cannot yet say which chromosome harbors the MAT gene in *A. nomius*, but the 65 kb contig that contains the *SLA2*, *MAT1-1* and *APN1* genes exhibits synteny to other section *Flavi* species and has sequence homology to chromosome VI in *A. oryzae*.

**Fig. 4:**
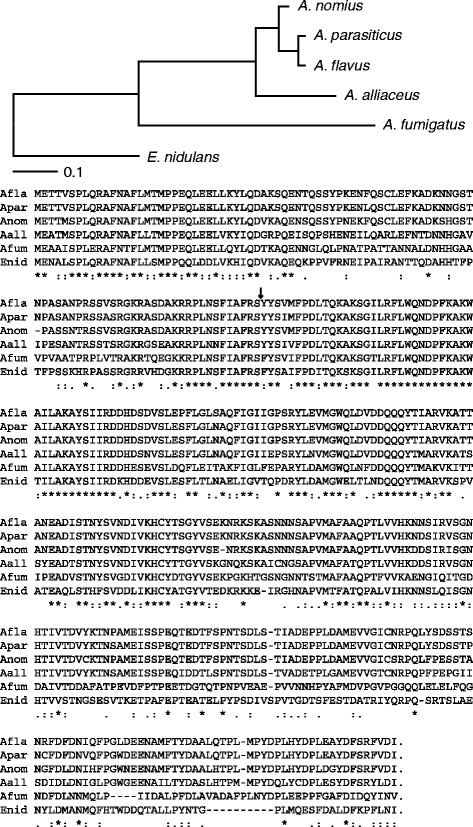
Phylogeny for *MAT1-1* idiomorphs of various *Aspergillus* species reveals patterns of ancestry for this locus. A maximum likelihood phylogeny was inferred for the *MAT1-1* gene of six *Aspergillus* species (top image). Also included is an amino acid alignment of the *MAT1-1* gene for the same six species that was generated using CLUSTAL-W (bottom image). Beneath each column are characters that relate to the level of conservation in amino acid substitutions among the different species. An asterisk (*) indicates that an identical amino acid residue exists for all species examined; a colon (:) indicates that at least one highly conserved amino acid substitution exists; a period (.) indicates that at least one semi-conserved substitution exists; and no character indicates that at least one un-conserved amino acid substitution exists. The black inverted arrow indicates the location of a 52 bp intron separating the two coding regions that comprise the *MAT1-1* gene in these organisms

Within section *Flavi*, comparison of the *A. nomius* amino acid sequence with other *MAT1-1* idiomorphs revealed 79 % identity to *A. alliaceus* and 91 % identity to both *A. flavus* and *A. parasiticus* (Fig. 4). Of the 374 amino acids aligned, 34 % were conserved, 32 % involved semi-conserved substitutions, and 34 % involved non-synonymous substitutions. The maximum likelihood (ML) tree, inferred from the *MAT1-1* amino acid alignment of six *Aspergillus* species, shows homothallic *E. nidulans* to be more basal to the other species, with homothallic *A. alliaceus* being the basal organism to *A. nomius*. However, this inference was based on a single locus. *Aspergillus alliaceus* was not included in our phylogenomic analysis because its genome has yet to be sequenced, so it is unknown whether or not this species would maintain its basal position compared to the rest of section *Flavi*.

Perhaps the evolutionary relationship of homothallic and heterothallic breeding systems can be inferred from this ML tree. For example, the *MAT1-1* ancestor to heterothallic Aspergilli diverged from *E. nidulans* and its remnant locus organization can be found in *A. alliaceus*, the only known *Aspergillus* species for which both MAT idiomorphs are adjacent to one other at a single locus [26]. Interestingly, *A. nomius* strains that have the *MAT1-1/MAT1-2* genotype are capable of bisexuality [4], but the chromosomal positioning of their *MAT* locus/loci has yet to be determined. Most strains of *A. flavus* and *A. parasiticus* are of a single mating type; however, some strains have been discovered to contain both genes (e.g., NRRL 3357) although only one of them is functional (Ignazio Carbone, personal communications). *Aspergillus nomius* strain NRRL 13137 contains only the *MAT1-1* gene, yet other strains have been discovered that contain both idiomorphs although incapable of self-fertilization [4]. Mapping the mating-type locus/loci in a bisexual strain of *A. nomius*, for comparison to other *MAT1-1/MAT1-2* strains, as well as inter-specific mating tests between *A. nomius* and closely-related species, should also be undertaken.

## Conclusions

Many avenues remain to be explored regarding this aflatoxigenic fungus. *Aspergillus nomius* appears to be the ancestral aflatoxigenic species based on genomic comparisons to other section *Flavi*. Future genomic comparisons should include more species within section *Flavi* as well as expand beyond section *Flavi* to encompass other aflatoxin-producing Aspergilli. Further comparisons are warranted for *A. nomius* strains that thrive in distinct ecological niches and that exhibit genomic differences. By elucidating its ecological, evolutionary and genomic differences, we may finally understand the reasons for speciation of other aflatoxigenic species as well as the purpose for aflatoxin production.

## Methods

### Sequencing the genome of *A. nomius* NRRL 13137

The genome of NRRL 13137 was sequenced using an Ion Torrent Personal Genome Machine (PGM) from Life Technologies (Grand Island, New York). To obtain genomic DNA, 75 ml of potato dextrose broth was inoculated with 500 μl of fungal spore suspension and placed in an orbital shaker set at 30 °C and 125 rpm overnight. Resulting mycelial pellets were then vacuum-filtered, with two sterile water washes, blot dried, and approximately 200 mg was used to extract DNA using a Masterpure Yeast DNA Purification kit (Epicentre Biotechnologies, Madison, Wisconsin). The template was first subjected to fragmentation using the Ion Xpress Plus Fragment Library kit. Template purification steps were performed using AgenCourt AmPure XP beads (Beckman Coulter, Inc., Indianapolis, Indiana) and a magnetic rack, followed by re-suspension with Low TE buffer. Size selection of DNA fragments was performed using the E-Gel system (Life Technologies) and library qualification was performed with an Agilent 2100 Bioanalyzer instrument and High Sensitivity DNA kit (Agilent Technologies, Santa Clara, California). A OneTouch 2 (OT2) instrument and OT2 400 kit (Life Technologies) were used to prepare template-positive ion sphere particles (ISPs). The ISPs were qualified using a Qubit fluorometer and then enriched using the Ion OneTouch ES system with its respective component of the OT2 400 kit (Life Technologies). Sequencing was performed using the Ion PGM Sequencing 400 kit, an Ion 318 Chip v2, and the PGM instrument (Life Technologies) following manufacturer’s instructions.

### Genome annotation

Newbler software [28] was used to assemble the *A. nomius* genome, and the annotations were created using the MAKER program [29]. MAKER produced *ab initio* gene predictions from the repeat-masked genomic sequence using Genemark [30] and Augustus [31]. Hint-based predictions were also generated from Augustus. Genemark was trained for *A. nomius* through its internal self-training module. An initial run of MAKER was performed to develop a set of genes that were used to train Augustus with predicted proteins from *A. oryzae* RIB40, *A. flavus* NRRL 3357 (L-type), *A. flavus* AF70 (S-type), and *A. parasiticus* SU-1 with the “protein2genome” option set. Both *A. oryzae* and *A. flavus* NRRL 3357 genome sequences and annotations were acquired from NCBI. The genome sequences and annotations for *A. flavus* AF70 and *A. parasiticus* were acquired from JCVI [32]. A subsequent run of MAKER was performed using Genemark and Augustus with the Uniref50 protein database [33] and the predicted proteins from *A. flavus* AF70 and *A. parasiticus* as evidence of protein homology. The predicted genes, from the second MAKER run, were then used to retrain Augustus, and MAKER was run a third time with the updated gene prediction file. Repetitive elements were identified within MAKER using the RepBase program [34] repeat library for fungi along with MAKER’s built-in repeat database known as RepeatMasker [35]. Functional annotation was added to the MAKER gene predictions using BLASTP against the NCBI fungal RefSeq protein database [36]. Gene names were transferred from the best BLAST hits if they had an e-value ≤ 1e–10, identity ≥ 50 %, and coverage ≥ 70 %. The genome annotation was converted to NCBI submission format using Genome Annotation Generator [37] and deposited at NCBI under BioSample #02768702.

### Genome comparison

Genomic comparisons between *A. nomius* and other Aspergilli; particularly those from section *Flavi*, were performed using various types of analyses. The Secondary Metabolite Unique Regions Finder (SMURF) and the Antibiotics & Secondary Metabolite Analysis Shell (antiSMASH) programs [38, 39] were used to predict secondary metabolite clusters in the five Aspergilli from section *Flavi* with sequenced genomes. SMURF and antiSMASH vary in the quantities of backbone genes they explore, so both were modeled to look for the same three backbone genes: PKS, NRPS and PKS-NRPS hybrid. We also performed a phylogenomic investigation, which first involved detecting orthologous proteins within other fungi using Proteinortho [40], aligning them using MUSCLE [41], and concatenating them into a 2.2 Mb amino acid alignment using GBLOCKS [42]. The phylogenetic tree was inferred using RAxML-HPC [43] with the rtREV amino acid substitution matrix and *Penicillium chrysogenum* as the outgroup organism. The protein sequences for *A. nidulans* and *P. chrysogenum* were retrieved from AspGD and JGI, respectively. To quantify the genes unique to *A. nomius* as well as those genes it shares with closely-related species from section *Flavi*, orthologs identified by Proteinortho were put into a Venn diagram using software found at [44].

### Gene ontology and function

GO terms were assigned to protein sequences within the BLAST2GO pipeline [45] using BLAST+ against the NCBI fungal database and InterProScan. A Fisher’s Exact test was run within BLAST2GO to identify GO terms that were over-represented among gene products unique to *A. nomius* with the reference set being GO terms for all non-unique *A. nomius* gene products. The data generated from the Fisher’s Exact test included only those GO terms that are enriched below a threshold of p ≤ 0.05, showed the percentage of sequences associated with each listed term, and ranked those terms from the highest to lowest level of enrichment.

### MAT locus investigation

A portion of the *MAT1-1* idiomorph for *A. nomius* NRRL 13137 had been previously sequenced and accessioned in GenBank (HQ001936). We then BLAST-queried the nucleotide sequence against our *A. nomius* genome and determined the respective contig for which we had 100 % identity and coverage, as well as a large span of sequence both upstream and downstream of our known sequence. Ramirez-Prado et al. [26] reported that there are conserved regions that flank the MAT idiomorph in certain Aspergilli. These regions include a gene for cytoskeleton assembly control (*SLA2*) and a gene for DNA lyase (*APN1*). Mapping of these loci, in relation to the idiomorph, involved the use of both nucleotide and amino acid sequences through BLAST query, and manual alignment using the Sequencher 5.4 program (Gene Codes Corporation, Ann Arbor, Michigan). Another component to analyzing the *MAT1-1* idiomorph for *A. nomius* involved using ClustalW [46] to create an amino acid alignment and to weigh substitutions for the same idiomorph across other *Aspergillus* species: *A. alliaceus*, *A. flavus*, *A. fumigatus*, *A. parasiticus*, and *E. nidulans*. Except for *A. nomius*, the protein sequences were obtained from the NCBI database. Additionally, a phylogenetic association was inferred for the *Aspergillus MAT1-1* idiomorph, based on the amino acids of these same species, using a software program known as Randomized Axelerated Maximum Likelihood [47].

## Abbreviations

NRRL: Northern Regional Research Laboratory
GO: Gene ontology
CZ: Czapek’s
CPA: Cyclopiazonic acid
OMST: O-methylsterigmatocystin
NCBI: National Center for Biotechnology Information
SMURF: Secondary metabolite unique regions finder
antiSMASH: Antibiotics & Secondary Metabolite Analysis Shell
BLAST: Basic local alignment search tool
MAT: Mating-type
bp: Base pairs
SLA2: Cytoskeleton assembly control gene
APN1: DNA lyase gene
ML: Maximum likelihood
PGM: Personal genome machine
JCVI: J. Craig Venter Institute
PSK: Polyketide synthase
NRPS: Non-ribosomal peptide synthase
RAxML: Randomized axelerated maximum likelihood
AspGD: Aspergillus genome database
JGI: Joint Genome Institute
